# Chalcogenide Glass Optical Waveguides for Infrared Biosensing

**DOI:** 10.3390/s90907398

**Published:** 2009-09-15

**Authors:** Marie-Laure Anne, Julie Keirsse, Virginie Nazabal, Koji Hyodo, Satoru Inoue, Catherine Boussard-Pledel, Hervé Lhermite, Joël Charrier, Kiyoyuki Yanakata, Olivier Loreal, Jenny Le Person, Florent Colas, Chantal Compère, Bruno Bureau

**Affiliations:** 1 Sciences Chimiques de Rennes, UMR 6226, Equipe Verres & Céramiques, Université Rennes 1, 35042 Rennes, France; E-Mail: Bruno.bureau@univ-rennes1.fr (B.B.); 2 Biomedical sensing and Imaging Group, Inst. for Human Science and Biomedical Engineering, National Institution of Advanced Industrial Science and Technology (AIST), Tsukuba, Japan; 3 National Institute for Materials Science, 1-1 Namiki, Tsukuba, 305-0041, Japan; 4 Institut d’Electronique et de Télécommunications de Rennes-Microelectronic, Université Rennes 1, 35042 Rennes, France; 5 FOTON-CCLO, UMR 6082-ENSSAT, Université Rennes 1, 22305 Lannion, France; 6 Dept. of Neurosurgery, Inst. of Clinical Medicine, University of Tsukuba, Tsukuba, Japan; 7 INSERM U522, IFR 140, University of Rennes 1, Rennes, France; 8 IFREMER, ERT-Service Interfaces et Capteurs, BP 70 29280 Plouzané, France

**Keywords:** chalcogenide, optical sensor, fibre, planar waveguide

## Abstract

Due to the remarkable properties of chalcogenide (Chg) glasses, Chg optical waveguides should play a significant role in the development of optical biosensors. This paper describes the fabrication and properties of chalcogenide fibres and planar waveguides. Using optical fibre transparent in the mid-infrared spectral range we have developed a biosensor that can collect information on whole metabolism alterations, rapidly and *in situ*. Thanks to this sensor it is possible to collect infrared spectra by remote spectroscopy, by simple contact with the sample. In this way, we tried to determine spectral modifications due, on the one hand, to cerebral metabolism alterations caused by a transient focal ischemia in the rat brain and, in the other hand, starvation in the mouse liver. We also applied a microdialysis method, a well known technique for *in vivo* brain metabolism studies, as reference. In the field of integrated microsensors, reactive ion etching was used to pattern rib waveguides between 2 and 300 μm wide. This technique was used to fabricate Y optical junctions for optical interconnections on chalcogenide amorphous films, which can potentially increase the sensitivity and stability of an optical micro-sensor. The first tests were also carried out to functionalise the Chg planar waveguides with the aim of using them as (bio)sensors.

## 1. Introduction

The use of chalcogenide glasses offers notable advantages such as remarkable optical properties like a wide transmission window (1–20 μm), depending on composition, high refractive indices, which allow a high portion of the light to be concentrated outside of the core material, making them suitable for sensitive detection of clinical or environmental changes [1–10]. They also present interesting non- linear optical properties, photorefractive effects, low phonon energies for active devices related to photoluminescence, not only explored on bulk glasses but also on fibres and planar waveguides (wavelength conversion, Raman and parametric amplification, laser sources for mid-IR ...) [11–19].

Emerging technologies related to thermal imaging as well as infrared sensors have prompted new research projects involving infrared transmitting materials, including chalcogenide glasses. The need for optical sensors operating in the mid IR region, where the main IR signatures of molecules and biomolecules are located, is playing an important role in the development of analytical techniques providing, for instance, *in-situ* information on metabolic mechanisms. For some 10 years now, infrared transmitting optical fibres have been especially designed to carry out a new spectroscopic technique called Fibre Evanescent Wave Spectroscopy (FEWS) [3,4,10,20]. To date pioneering works have been carried out involving numerous partners from various domains within the framework of multidisciplinary research programs. In this paper, some results concerning medical applications will be presented.

Among the many fields where integrated optics may be applicable, the development of optical sensors is one the most promising, leading to low cost and highly compact optical systems. Slab waveguides based on chalcogenide amorphous films with good adherence and controlled composition that can include rare earths may be obtained using different modes of deposition such as thermal evaporation [21–25], chemical vapour deposition [26] pulsed laser deposition [27–30] and RF-sputtering [31–33]. The next step has been to produce rib waveguides, i.e., one-dimensional guiding structures composed by a thin high-index layer deposited on a low-index substrate. Lithography and plasma etching are standard patterning techniques used in microelectronics to design and manufacture complex optical functions on waveguides within a small and compact chip format. The aim of this work was to show the possibility of obtaining sulphide waveguides with good physical properties and low losses using RF-magnetron sputtering for deposition of thin films and dry etching to make rib waveguides. Thus, an optimization of these films and their dry etching will arise from the characterization of their morphological, topographical or optical properties. The characterization of these optical waveguides is an essential step for the proper development of these materials for applications in the optical sensor field.

## 2. Results and Discussion

### 2.1. Fibre as Useful Tool for Detection

#### 2.1.1. Set Up- Detection by infrared fibre evanescent wave spectroscopy (FEWS)

**Figure 1.**
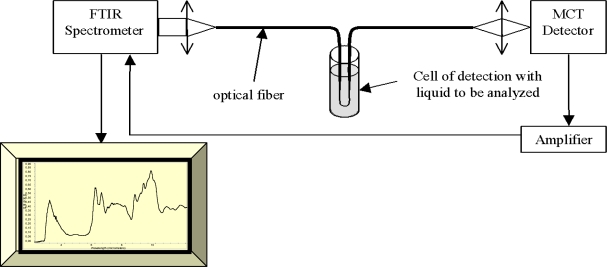
Schematic representation of the experimental set up used for FEWS experiments. It is composed of an IR source, a chalcogenide glass.

**Figure 2.**
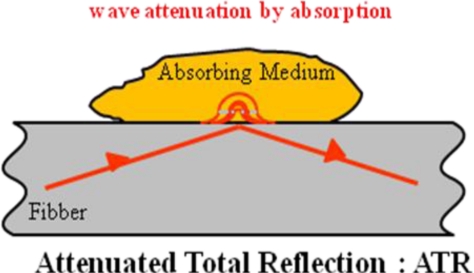
Schematic representation of the experimental set up used for FEWS experiments.

Fibre Evanescent Wave Spectroscopy is an efficient and easy way to record infrared spectra. FEWS enables *in situ* and in real time studies with no sampling. As represented in Figure 1, the experimental setup consists of a Fourier Transform Infra Red (FTIR) spectrophotometer coupled with a chalcogenide glass fiber and a mercury cadmium tellurium (MCT) detector. The principle of evanescent wave spectroscopy is based on the fact that the light propagates in the optical fiber by total reflections at the interface between the glass and the air. At each reflection, a part of the energy is absorbed by any chemical or biological species having absorption bands in the IR spectral zones (see Figure 2). Thus, the signal picked up at the fiber output corresponds to the initial signal minus the absorbed signal giving rise to the spectrum.

The optical fibre sensor used in this study was made of chalcogenide glass from the Te-As-Se (TAS) glass family. This glass is characterized by a high refractive index (around n = 2.8 from 2 to 12 µm), a large transmission window in the MIR, from 2 to 16 μm. Moreover, its thermo-mechanical properties make it easy to shape into optical fibres by pulling a glass rod at high temperature (see Section 3.1). To increase the level of detection, a tapered part is created and used as a sensing zone. Typically, the profile in diameter is 400 μm in the transportation section and 100 μm in the tapered section. In this experimental configuration, the evanescent wave penetration depth allows probing of only the very first microns of the sample. The sensing part of the fibre is maintained in a U-shape and directly put in contact with the biological sample, and then, a spectrum is collected. A background spectrum is collected before each experiment. Spectral resolution is set to 4 cm^−1^ and spectra resulted from the co-addition of about 100 scans.

#### 2.1.2. Examples of sensing

##### a. Metabolism alterations during cerebral ischemia in rat model

A surgical transient focal-cerebral ischemia was produced on the right hemisphere of rat brains. For this purpose anaesthesia was induced with an intra-peritoneal injection of a mixture of ketamine and xylazine. After induction, the animals were ventilated thought a facemask with 2% isoflurene in a mixture of 30% O_2_ and 70% N_2_. The body temperature was maintained at 37°C by a heating pad. Heart rate and pO_2_ were monitored with a pulse oximeter (Nonin 8600MV). Transient focal ischemia in the area perfused by middle cerebral artery (MCA) was induced as follows: briefly, the occluder, a 4-0 nylon suture with a silicone-coated tip (0.25 mm in diameter), was advanced from the external carotid artery into the lumen of the internal carotid artery until it blocked the origin of the MCA. Reperfusion was accomplished by withdrawal of the suture. Ischemia was performed during 60 min, following by a period of 120 min of reperfusion before sacrifice.

A few millimetre slice of each brain sample is used for the fibre experiment. The slice chosen encompassed a part of the damaged zone, as determined by histological staining. Then, the fibre is put in contact with different parts of the brain slice, sub-cortical zone of each hemisphere. The contact zone between the fibre and the brain sample is about 1 mm.

**Figure 3.**
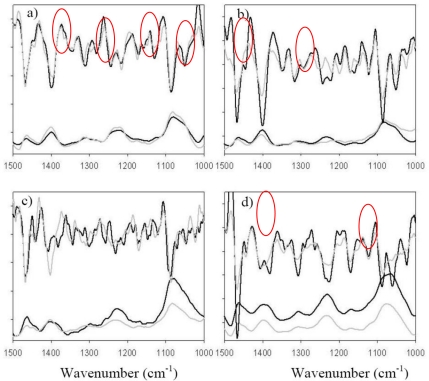
IR spectra and second derivative of the four brain samples analyzed. Black spectra and derivatives correspond to the left (normal) hemisphere. Grey spectra and derivatives correspond to the right (ischemic) hemisphere. The circles denote the relevant spectral areas as explained in the text.

Thanks to the optical fibre sensor, the sub-cortical area of four rat brains was analyzed by remote MIR spectroscopy. The left and right hemisphere MIR spectra from a given coronal slice were compared for each brain sample (Figure 3). The left hemisphere was considered the control one. Indeed, transient focal ischemia was provoked in the right hemisphere and histological staining using 2,3,5-triphenyltetrazolium chloride (TTC) did not reveal any damaged areas. The right hemisphere spectra of each rat brain exhibited spectral differences in comparison with left hemisphere spectra of the same sample. By analysing the second derivative spectra, the main spectral differences have been assigned for the four brain samples.

For rat brain n° 1 (Figure 3a), differences on the right hemisphere spectrum could be attributed to cerebroside (1,049 cm^−1^), peptidoglycan (1,157 and 1,129 cm^−1^), proteins (around 1,300 cm^−1^) and amino acids (1,444 cm^−1^). For brain n° 2 (Figure 3b), main differences were attributed to amino acids (1,444 cm^−1^), amide III (characteristic of proteins around 1,250 cm^−1^) and phosphodiester. For brain n° 3 (Figure 3c), the right hemisphere spectrum was really different in comparison with the left hemisphere one. By analyzing the second derivative, spectral differences appear in the region between 1,000 and 1,400 cm^−1^ and correspond to a lot of metabolites. Brain metabolism was very affected by the ischemia and reperfusion. For the brain sample n° 4 (Figure 3d), some differences were noticed between the two hemispheres. They were attributed to digalactosyl (1,059 cm^−1^) and amino acids (1,381 cm^−1^). The absorbance level for right hemisphere of n° 3 and n° 4 sample was less significant than in the left one. This could be attributed to a decrease of the metabolism activity in the ischemic hemisphere, due to a lack of oxygen during ischemia [34,35].

##### b. Mouse serum analysis

Animals used in this study were four mice reported to develop obesity related to an homozygous mutation in the leptin gene, leading to hyperphagia and type II diabetes. In addition four mice, which are heterozygous for the mutation and do not develop obesity, were used as control. All animals were maintained in accordance with French law and regulations. After anaesthesia blood samples were obtained by intracardiac puncture and serum was then separated by centrifugation after coagulation. Fresh serum was used for infrared spectroscopy. In addition, glycaemia (expressed as mean ± SD) was quantified using an enzymatic method (glucose oxidase). For infrared analysis, 10 μL of serum was put down on the fiber. Then the fibre was turn up and the spectrum then acquired. Two spectra were obtained from each sample. Note that the spectra shown in Figure 8a are recorded by bringing a serum drop in contact with the fibre. So the length of contact is no more than few millimetres. Principal Component Analysis (PCA) was performed on the spectra using the BRUKER, OPUS Ident software. This unsupervised method allows extraction of information on analysed samples from their infrared spectra without explicitly attributing the absorption bands to the vibrationnal bonds.

The PCA consists in considering the initial spectra as linear combinations of so-called factor spectra. The significance of a particular factor spectrum is given by the corresponding coefficient of the combination, called score. The software arranges directly the factor spectra according to these scores: the first one is the most significant and the higher indexed ones quickly represent very noisy factor spectra. The PCA map represents the initial spectra as points in two or three dimension spaces. The axis corresponds to the factor spectra and the co-ordinates are the corresponding scores. Generally, the first factor spectra of the combination are the more relevant to discriminate the initial spectra into non-overlapping clusters. Note that generally the PCA is not implemented for the whole infrared spectra, but rather restricted to frequency ranges which present large inter-sample variances.

**Figure 4.**
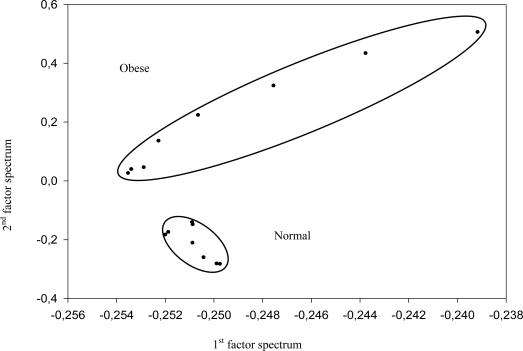
PCA map showing two distinct zones where appears the control spectra on one hand, and the obese spectra on the other hand.

No visible spectral signature, like line shifts or variation in intensities, enabled differentiation between both families. Then PCA was implemented in the 1,100–1,000 cm^−1^ range corresponding to the sugar rings vibration bands. The PCA map obtained by considering the two first factor spectra is presented in Figure 4. The points corresponding to the control spectra were well grouped, showing that the spectra are very homogeneous. This result is consistent with the fact that glycaemia in all these animals were similar (10.7 ± 1.1 mmol/L). On the other hand, the obese spectra were localised in a totally different area. This point could be related to the high level of glycemia found in those animals (32.6 ± 3.7 mmol/L). However, the spectra from obese mice were spread over a larger zone. This point remains to be understood. Taken collectively our results demonstrate that FEWS experiment coupled with unsupervised methods of analysis like PCA is an efficient tool to distinguish serum from obese and control mice.

### 2.2. Chalcogenide Planar Waveguide for Sensing Application

#### 2.2.1. Planar waveguide fabrication

**Figure 5.**
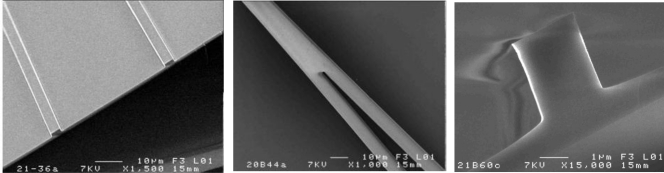
SEM micrographs showing waveguide cross section and Y junction rib waveguide made of Ge_25_Sb_10_S_65_ deposited by sputtering.

The difficulties of manufacturing micron-and nano-size structures in chalcogenides compared to oxide glass or SOI are well-known [36–38]. Germanium based chalcogenide glasses were selected for their robustness, expected with presence of germanium atoms and the relatively high Tg for chalcogenide glasses. The physical properties of these chalcogenide films turned out to be suitable for RIE etching. By optimizing the pure CF_4_ plasma process parameters (power, gas pressure and gas flow rate), an anisotropic etching behaviour was obtained with a reasonable etching rate equal to 300 and 70 nm·min^−1^ for Ge_25_Sb_10_S_65_ and Ge_25_Sb_10_Se_65_ sputtered films, respectively, and with an overcut angle (positively sloped sidewall) under 10°. The use of oxygen can be avoided in our case and this allowed a better control of the composition of the chalcogenide film limiting the formation of oxide film by oxygen diffusion. A single mode fibre was used to couple light in the waveguides and the external field excited different modes. Although the simulation showed that these rib waveguides supported multiple modes at 1,550 nm, higher order modes generally presented high losses due to the stronger coupling of the propagating field in relation to the sidewalls. As a result, the measured loss values include losses by several modes, notably the surface scattering losses at different excited modes. For chalcogenide waveguides, values of 0.5–1 dB/cm were obtained and are principally due to the contribution of surface scattering at different excited modes. Similar loss values of the same order of magnitude were obtained using multimode chalcogenide glass rib optical waveguides [8,37,38]. Waveguide splitters are important elements in a variety of applications, like power splitters for planar lightwave circuits or optical sources for integrated microfluidic devices [39]. The sensitivity and stability of optical sensors can be improved by using a system based on a Y junction where one arm can be used as reference beam. Finally, straight Y junctions with a guide width of 2 μm were formed on sulphide or selenide films by similar processes. Figure 5 shows different SEM images of a waveguide cross section and Y junction rib waveguide made of Ge_25_Sb_10_S_65_ deposited by sputtering, respectively. The Y junction structure was designed in order to be singlemode or very weakly multimode with a film thickness of 500 nm and 4 μm width at the input and 2 μm after the Y junction in order to allow a good partition in the two branches and also to improve the evanescent field extended into the media surrounding the waveguide. The excess loss expected for such structures due to the separation of branches has been evaluated around 2–3dB permitting transmission of the light with controlled optical losses. Following these first results, detection experiments with these micro-structures are in progress.

#### 2.2.2. Biofunctionalisation of sulphide films

A change in the optical characteristics of the external environment (i.e., a change in refractive index or a biochemical reaction) involves a modification of the properties of optical propagation in the waveguide. This phenomenon is induced via the evanescent field spreads over several hundred nanometers into the external environment. For the evaluation of specific interactions, the receiver is attached covalently on the sensor surface, while the complementary molecule is free to come onto the receiver. The recognition of the complementary molecule by the receiver will cause the change of refractive index detectable by the optical sensor.

**Figure 6.**
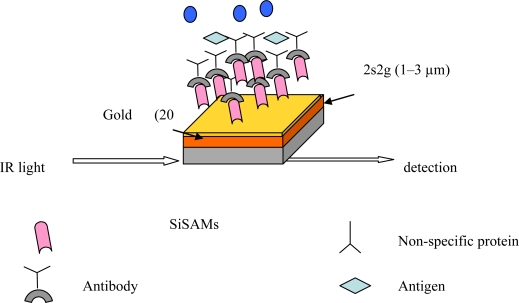
A schematic view of a functionalised 2s2g film.

Considering the use of chalcogenide film as biosensor, an attempt at biofunctionalisation of these films was carried out on planar waveguides. One of the most useful techniques to prepare layers is the formation of self-assembled-monolayers (SAMs) [40]. Due to difficulty posed by chalcogenide chemistry like their high reactivity with basic solvents, functionalisation through gold-chemistry was chosen. Gold is a convenient metal because of its small dielectric constant and its chemical durability towards oxidation, which is important for biological applications. A 20 nm-gold layer was deposited by rf sputtering on the top of the sulphide waveguide (Figure 6).

**Figure 7.**
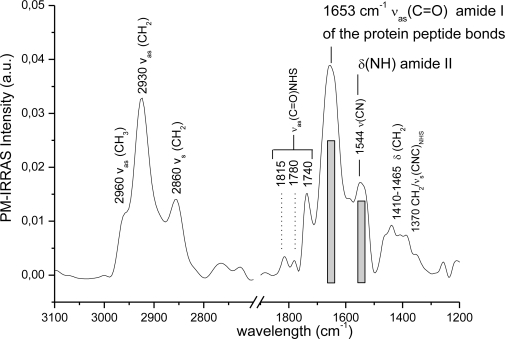
PM-IRRAS analysis of the planar waveguides functionalized after reaction with MUA, NHS/EDC and avidin.

We have observed a good adherence of gold on the surface of the films which can’t be easily scratched or peeled off and might be due to the creation of Au-S bonds. Anchor molecules (1,1 mercaptoundecanoic acid, MUA) were hooked on the gold to form SAMs on which antibodies could be attached. SAMs was activated by NHS/EDC (*N*-hydroxysuccinimide, 1-ethyl-3-carbodiimide hydrochloride) in order to form amide bonds which act as go-between SAMs and the capturing agents (i.e., antibodies). The attachment of the SAMs, NHS/EDC and a protein like avidin was confirmed by PM-IRRAS analysis. More precisely, the presence of amide I and II groups at 1,653 cm^−1^ and 1,544 cm^−1^, respectively, is characteristic of linked avidin (Figure 7). The quality of the sulphide film was not damaged, as was confirmed by SEM analysis of the film surface and cross section at the end of the chemical process. The first steps of functionalisation of planar chalcogenide waveguide were demonstrated and allow envisaging their use as an IR biosensor.

## 3. Experimental Section

### 3.1. Synthesis of Glass Targets and Preforms, Fibber and Planar Waveguide Fabrication

**Figure 8.**
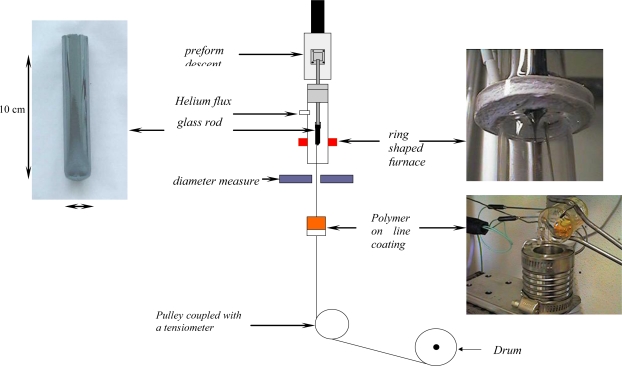
Schematic representation of the drawing tower. From a selenide glass rod photographed on the left, we obtain typically 25 m of fibre with a 400 μm diameter. Note that we can apply a coating polymer on-line to improve the mechanical behavior of the fibres.

Chalcogenide glass targets and preforms were synthesized from commercial elemental precursors (Ge, Sb, As, S, Se, Te) of high purity (5N). The precursors, despite their high purity, present surface oxidation and can be polluted by water. Elements as As, S, Se, and Te were specially purified to remove oxygen, molecular water, carbon and silica. These impurities introduce absorption in the mid- and far-IR ranges, as well as bulk scattering losses due to micro-inclusions. The chemical reagents S and Se were purified by vacuum distillation with a low rate of evaporation. All the elements were placed in evacuated quartz ampoules and then sealed. The synthesis was performed in a rocking furnace in the temperature range of 650–800 °C for 8–12 h, depending on compositions. The ampoules with the melt was then air-quenched. Targets and performs were directly obtained from glass rods in the form of cylinders of 15 or 50 mm in diameter. The drawing process is carried out using a fibering tower specially adapted for chalcogenide glasses (Figure 8).

Chalcogenide films were deposited by RF-magnetron sputtering on top of surface oxidized silicon substrates. Deposition was carried out at a working pressure in the range of 5 × 10^−3^–5 × 10^−2^ mbar. The sputtering power was maintained at low RF power (15–50W) during the deposition. The layer and rib waveguide surface were analysed by field emission scanning electron microscopy (JEOL JSM 6301F).

A classical photolithography process was used to prepare dry etching with a positive Shipley S1813 photoresist. First, a 1.3 μm thick photoresist was spin coated on the wafers. After softbake, the photoresist was then exposed to UV by means of a Cr-mask using a Suss Microtech MJB3 i-line mask aligner. The exposed bands were dissolved with a commercial developer. Considering the high reactivity of chalcogenide films in relation to the NH_4_OH or KOH-based developer, the TMAH-based developer Microposit MF 319 was selected. After hard-bake, different parameters were investigated in the etch process, gas flow from 1 to 30 sccm, gas pressure between 0.3 and 30 mTorr and RF power in the range of 30–300 W. CF_4_ gas was selected since the F^−^ ions could form volatile compounds with sulphide or selenide. The etch rates were determined by SEM on film cross-sections. The effective refractive indices of the propagation modes in the planar waveguide were measured with a Metricon-2010. Four laser beam wavelengths in the range from 633 to 1550 nm for both TE and TM polarization and rutile prism were used to excite the TE and TM modes inside the planar chalcogenide waveguides. Optical losses were measured by studying the scattered light from the surface of the waveguides. These waveguides varied in length from 2 to 2.5 cm. The laser light was single-mode fibber-coupled into the waveguide. The intensity of the scattered light was recorded with a digital camera placed above the sample. Transverse scanning along the light propagation direction enabled us to obtain the 2-D light intensity distribution of the waveguide modes. The longitudinal variation was obtained by integrating the data along each sampling transverse line. The light intensity decreased exponentially with the z-propagation distance. In this study, the attenuation values were the average of several measurements performed on several samples. The polarization of the coupled light in the waveguide was not controlled. In addition, light propagation was observed at the output of the waveguides by near field profiles of guided modes at 1,550 nm.

## 4. Conclusions

Chalcogenide glasses are a matchless material exhibiting large optical transparency in the infrared range, together with thermo-mechanical properties that make them easy to shape into optical devices such as lenses, fibres, or planar guides. In that context, the development of optical sensors is surely one of the most exciting and promising uses for these chalcogenide materials. In that framework, optical fibre and a planar waveguide have been successfully designed, exhibiting suitable properties for use as infrared sensors. Some pioneer FEWS works, performed with a fibre on rat or mice, have already revealed all the potential of such systems, especially in the medical field. One of the next steps will consist in emphasizing our effort on the statistical tool in order to pull out all the relevant contents of such spectra.
